# Effects of Ugali Maize Flour Fortification with Chia Seeds (*Salvia hispanica* L.) on Its Physico-Chemical Properties and Consumer Acceptability

**DOI:** 10.3390/foods13040543

**Published:** 2024-02-09

**Authors:** Susan Chemutai, Monica Mburu, Daniel Njoroge, Viktoria Zettel

**Affiliations:** 1 Institute of Food Bio-Resources Technology, Dedan Kimathi University of Technology, Nyeri-Mweiga Road, Private Bag, Dedan Kimathi, Nyeri 10143, Kenya; susanchemutai99@gmail.com (S.C.); daniel.njoroge@dkut.ac.ke (D.N.); 2 Department of Process Analytics and Cereal Science, Institute of Food Science and Biotechnology, University of Hohenheim, 70593 Stuttgart, Germany; viktoria.zettel@uni-hohenheim.de

**Keywords:** ugali, whole maize meal, defatted chia flour, whole chia seeds

## Abstract

The study investigated the effect of incorporating whole chia seeds (WCS) and defatted chia seed flour (DCF) into whole maize meal for ugali preparation. Both were incorporated at substitution levels of 3%, 6%, and 9% separately, and the resulting treatments subjected to laboratory analysis. In addition, ugali samples were prepared from all the resulting flour formulations and subjected to consumer acceptability assessment. Incorporation of both DCF and WCS resulted in increased water absorption capacity (ranging from 0.78 to 0.98 g/mL), swelling index (ranging from 0.15 to 3.25 mL/g), and swelling capacity (ranging from 2.46 to 5.74 g/g). WCS decreased the bulk density and oil absorption capacity. DCF, however, resulted in an increase in bulk density and oil absorption capacity. Both DCF and WCS lowered the lightness (L*) of the products. Proximate composition ranged from 4.78 to 7.46% for crude fat, 7.22% to 9.16% for crude protein, and 1.74 to 4.27% for crude fiber. The obtained results show the potential of chia seeds as a good fortificant of maize flour since it resulted in nutritionally superior products (crude ash, crude protein, crude fat, and energy value) when compared to control. The freshly prepared ugali samples were generally acceptable to the panelists up to 9% WCS and 6% DCF substitution levels.

## 1. Introduction

Malnutrition is a nutritional state that involves deficiency/excess of energy, proteins, or micronutrients, harming the form and function of tissue/body, sometimes meaning a clinical outcome [1,2]. The standard conditions of undernutrition in African countries are protein energy malnutrition (PEM) and micro-nutrient deficiency (MND), with effects that could be severe or mild [3]. According to a relevant study, 23.8% of the sub-Saharan Africa population suffered malnutrition between the years 2012 and 2014 [4]. Topping on the insufficiency of food, the staple foods consumed are starchy, and they are deficient in proteins with little or no source of other macro and micronutrients. Thus, overreliance on staple foods like maize to provide one with all the required nutrients is majorly the cause of the rampant PEM and dietary MND [5]. Nutrition intervention include food fortification policies and non-food-based strategic programs like supplementation and dietary diversification, where food-based strategies have proven attractive options for improving micronutrient uptake [1].

Maize (*Zea mays*) flour remains one of the leading staple food in Kenya, consumed in various forms like porridges and ugali (stiff porridge) [6]. White maize commonly used in Kenya is low in protein and minerals like zinc, potassium, calcium, and B-complex vitamins [7]. However, ugali is consumed by 70% of the population in Kenya, primarily for main meals [8]. Since the protein sources of side dishes taken with ugali are expensive, a significant part of the population consumes it with green leafy cheaper vegetables, thus, increasing the rate of PEM [9]. Fortification of this staple food could be an effective option for reaching most of the population. The government of Kenya has put in place a requirement for all maize millers to fortify their flours with minerals and vitamins. However, it was reported that only 37% of the maize meal brands have adopted this plan [10]. The low adoption has led to efforts by researchers to come up with food-to-food fortification options to provide a long-term solution that reaches all, including those in rural areas [6].

Chia (*Salvia hispanica* L.) is an annual herbaceous plant of the *Lamiaceae* (mint) family that is rich in oil. It has been termed a superfood since it is high in polyunsaturated fatty acids (PUFA), minerals, proteins, antioxidants, and vitamins, hence, it is used for nutritional [11] and health benefits [12]. In recent years, this functional food has been used as a food additive to improve food quality [13,14]. This pseudocereal is underutilized, yet when compared to other cereals like amaranth, wheat, rice, barley, oats, and maize, it is higher in protein content by about 15 to 23% [15]. The seeds are an excellent source of omega-3 PUFAs ranging from 58 to 64% of total lipids and 16 to 24% of proteins [16]. As a result, incorporating chia seeds into new functional foods is a potential and novel method of incorporating omega 3 and omega 6 fatty acids into food products [17]. Furthermore, polyphenols found in abundance in chia seeds have antioxidant properties and are crucial in preventing several chronic illnesses. They are also beneficial for those with celiac disease because chia seeds do not contain gluten [15]. Pasta, cakes, bread, and biscuits are a few food products that have been incorporated with chia seeds in other studies [18]. Chia seed flour has also been used in developing cassava porridge flour blends [19,20]. This study aimed at fortifying whole maize meal with defatted chia seeds flour and whole chia seeds and, hence, examine their effect on functional, color, and pasting properties, as well as the subsequent effect on the proximate composition and consumer acceptability of the resulting ugali products.

## 2. Materials and Methods

### 2.1. Samples Preparation

Maize seeds were purchased from Nyeri town market, Kenya while chia seeds were obtained from Dedan Kimathi University of Technology chia demo farm, Nyeri, Kenya. The maize seeds were then sorted and dried using solar drier to a moisture content less than 10% before being milled into flour. To obtain the defatted chia flour, cold-pressing method was used to extract the oil using the procedure described by [21] and the de-oiled fraction was subsequently milled (chia seeds cake). Milling of maize and de-oiled fraction of chia was performed separately using the ultra-centrifugal machine (ZM 200, Retsch GMbH, Haan, Germany) through 1.00 mm sieve at 8000 rpm. Using defatted chia flour (DCF), different flour formulations were prepared using 3%, 6%, and 9% substitution levels in whole maize meal and labeled as 3% DCF, 6% DCF, and 9% DCF. The same was performed using whole chia seeds (WCS) and labelled as 3% WCS, 6% WCS, and 9% WCS. A sample containing 100% whole maize meal was used as control. All samples were then subjected to analysis in triplicates.

To prepare ugali for consumer acceptability test, water and formulated flour were weighed at a ratio of 1:2.5 (flour to water). Water was first brought to boiling point in an aluminum cooking pot. The flour was then gradually added while constantly stirring under medium heat using a wooden cooking stick for around 7 min. The ugali was then turned and left to cook under low heat for another three minutes then served while hot.

### 2.2. Functional Properties

The following functional properties were determined: bulk density, water absorption capacity (WAC), oil absorption capacity (OAC), swelling index (SI), and swelling capacity (SC). Specifically, 50 g of the sample was weighed into 250 mL measuring cylinder and gently tapped on the surface of the bench from about 10 cm height up until a steady volume was attained. The final volume of the flour was then recorded, and bulk densities determined by dividing the sample’s weight by its volume then expressed as g/mL [22]. The ability to absorb water (WAC) and oil (OAC) was determined by mixing 1 g of flour formulation with 10 mL distilled water and vegetable oil, respectively, for a minute then allowing the mixture to stand at ambient temperatures for 30 min. Centrifugation (Hettich Zentrifugen, D-78532 Tuttlingen, Germany) at 3000 rpm was then performed for 30 min. After obtaining the volume of the supernatant, the water and oil absorption capabilities were computed as the difference between the volume of the supernatant and the original volume of water or oil applied to the flour and expressed as mg/mL [23]. 

Swelling index (SI) and swelling capacity (SC) were determined as per the method described by [24] with slight modification. Three grams of the sample were placed in a graduated falcon tube and 10 mL of distilled water added to it. The mixture was then vortexed for a minute and left to settle, then the volume was recorded. Subsequently, they were allowed to stand for one hour at room temperature, and then the new volume of the gel was recorded. The wet gel was then weighed using analytical balance (ENTRIS (224I) Göttingen, Germany) and recorded. The SI and SC were then calculated as follows:

(1) $$SI=\frac{Volume\;after\;soaking-Volume\;before\;soaking}{Sample\;weight}$$

(2) $$SC=\frac{Wet\;gel\;weight}{Sample\;weight}$$

The color of the flour formulations was measured using a Minolta Chroma-meter (CR-410, Konica Minolta, Osaka, Japan) in the L* a* b* color space (CIELAB). Dark to light (0–100) are visualized as lightness, or the L* value. The redness (a*) value determines how much of a red–green color is present (redness is +a* while greenness is −a*). The b* (yellowness) value describes how yellow–blue the color is to a given extent (+b* = yellowness and −b* = blueness). Calculated using (a*^2^ + b*^2^)^0.5^, Chroma is a measure of a color’s purity or saturation. (Tan ^−1^(b*/a*)) (180/π) +180 was used to determine the hue value, which quantifies the color’s most noticeable value [25]. Total color difference was calculated using the formula: E* = (L*^2^ + a*^2^ + b*^2^)^0.5^ [26] where the standard values on white plate for L*, a*, and b* were 98.8, −0.2, and 1.8. respectively.

### 2.3. Pasting Properties

The pasting properties of the flour samples were evaluated using standard procedure 1 using a Rapid Visco-Analyzer (RVA-4 Standalone, Newport Scientific, Warriewood, Australia) in compliance with AACC method 76-21.02 [27]. The software Thermocline (TCW3) version 2.6 was used to operate the RVA equipment. Suspensions of flour and water were maintained at 50 °C for one minute, heated to 95 °C for ten minutes, and then cooled to 50 °C for two minutes. The amount of distilled water added, along with the sample weights, were calculated based on a moisture content of 14%. Different flours’ pasting properties were determined using suspensions prepared from 3 g flour sample and 25 g distilled water (28 g total weight). The measurements recorded by RVA during studies comprised peak, trough, breakdown, final, and setback viscosities (all in mPa s), peak time (in minutes), and pasting temperature (in degrees Celsius). Three sets of determinations were made [27].

An automatic electronic moisture analyzer (KERN & Sohn GmbH, MLS SO- 3D, Balingen, Germany) was used to determine the moisture content of the flour samples. Using the procedures outlined in EU Commission regulation (EC) No. 152/2009-parts M, H (Soxhlet, solvent extraction), C (Kjedahl), and I (Fibretherm), examination of crude ash, crude fat, crude protein, and crude fiber were determined, respectively, by analytical chemistry module of the core facility. Total carbohydrates were calculated using the difference method, as described by [7] and energy values determined using Atwater’s conversion factors of 4, 4, and 9 for crude protein, carbohydrates, and crude fat, respectively as described by [28].

### 2.4. Sensory Evaluation

Both male and female panelists that were over 18 years of age and regular consumers of ugali were recruited to determine consumer acceptability of ugali samples made from chia-seed--fortified flours using completely randomized design. The panelists were instructed to maintain silence during the exercise and use the provided water and spittoon to rinse their palates between samples tasting. They were then provided with a score card with the respective codes of samples where they were asked to rate the liking of the products’ color, aroma, taste, mouthfeel, and overall acceptability using a 9-point hedonic scale in which 9 = ‘like extremely’ while 1 = ‘dislike extremely’. The samples from non-fortified maize flour that served as control were served first to prevent first order bias [6].

### 2.5. Statistical Evaluation

The effect of defatted chia seed flour and whole chia seeds on maize flour physicochemical properties and consumer acceptability of ugali was tested using a one-way ANOVA using Minitab Release 18 software (Minitab Inc., State College, PA, USA). Separation of means was performed using Fisher pairwise LSD method at 95% confidence interval (*p* < 0.05). 

## 3. Results and Discussion

### 3.1. Functional Properties of Maize Flour Fortified with Chia Seeds and Defatted Chia Cake Flour

Functional properties involve any property of the food that modifies or affect some of its characteristics and contribute to the final product quality [29]. Bulk density reflects the flour’s heaviness and a sign of the porosity of the product and is dependent on the particle size [23]. As shown in Table 1, addition of DCF at all levels increased the bulk density where 9%, had the highest value of 0.83 g/mL. The addition of WCS, however, resulted in a decrease in bulk density, where the formulation that had 9%WCS recorded the lowest bulk density of 0.72 g/mL, significantly different from control. This could be explained by the increased porosity and particle size in flours with incorporated WCS, thus, a higher volume and vice versa for samples with DCF [23]. Bulk density of whole maize meal as observed in this study was 0.79 g/mL, which was dissimilar to that reported by [29]. WAC of the flour formulations ranged between 0.78–0.98 mL/g. The addition of both WCS and DCF resulted in an increased WAC with significant difference from control at *p* < 0.05. This may be explained by the impact of bioactive substances that chia seeds possess and have been reported to have a high water absorption capacity [30]. Other factors that could cause enhanced water absorption capacity include high protein content and high fiber content [31].

Oil absorption capacity (OAC) observed in this study ranged from 0.22 to 0.67 mL/g. As compared to control at *p* < 0.05, the fortification at 9% level DCF significantly increased the OAC to 0.67 mL/g, while WCS significantly decreased the OAC to 0.22 mL/g. As explained by [32], OAC is the measure of the ability of oil to bind proteins’ hydrophobic (non-polar) sides and a higher number of these non-polar groups of the protein near the surface hold more oil and are associated with mouthfeel enhancement while retaining food flavor [33]. The observation made in this study could be attributed to this, since the defatting and milling of chia seeds cake into flour could have led to more exposure of these groups of protein near the surface compared to whole chia seeds that could have been less exposed.

The observed SI ranged from 0.15–3.25 mL/g while the SC ranged from 2.46 to 5.74 g/g. The flours that were fortified with both defatted chia seeds and whole chia seeds at all levels had significantly higher SI and also increased SC compared to control when compared at *p* < 0.05, where DCF had a higher impact compared to WCS. This could be attributed to the mucilage in chia seeds that is secreted when chia seeds encounter water, resulting in the generation of a high viscosity solution. The defatted chia seeds flour had a higher impact than whole chia seeds. This could be attributed to higher fiber content in defatted chia seeds flour compared to whole chia seeds.

### 3.2. Color

According to [34], L* represents lightness (pure black at 0 and pure white at 100), Hue is represented by a* on a green (−a*) to red (+a*) axis, and by b* on a blue (−b*) to yellow (+b*) axis in relation to a white reference. Color change from an ordinary one is known to affect consumer perception and acceptance [35]. Table 2 shows the effect that chia seeds had on the color properties of maize meal.

L* values for the different formulations of flours ranged from 85.34 to 93.23 where control had the highest value. At *p* < 0.05, both chia seeds and defatted chia flour addition significantly decreased lightness of the flour, where 9% level of defatted chia flour scored the lowest. The green color (a*) predominance significantly increased with addition of both defatted and whole chia seeds. The predominance of the yellow color (b*) was significantly lowered for all the samples with added defatted and whole chia seeds. The hue angle of the flour formulation ranged from 92.86 to 96.82. This denotes a color shift from yellow towards green. Thus, all the samples had a lawn color [36]. Chroma was significantly lower for all the flour formulations when compared to control at *p* < 0.05 except the formulation with 3% whole chia seeds, which was not significantly different from control. This explains the green color added to the flour samples by chia seeds and defatted chia seeds, which masked the yellow color while the two color blends reduced the brightness of a single color. A similar trend was observed by [37], where chia seeds reduced both the lightness and yellowness of wheat bread. It was noted that incorporation of DCF resulted in a significant difference in total color difference (∆E*) when compared to control at *p* < 0.05 at all levels of substitution. However, incorporation of WCS did not have significant influence on total color difference when compared to control.

### 3.3. Pasting Properties

**Figure 1:**
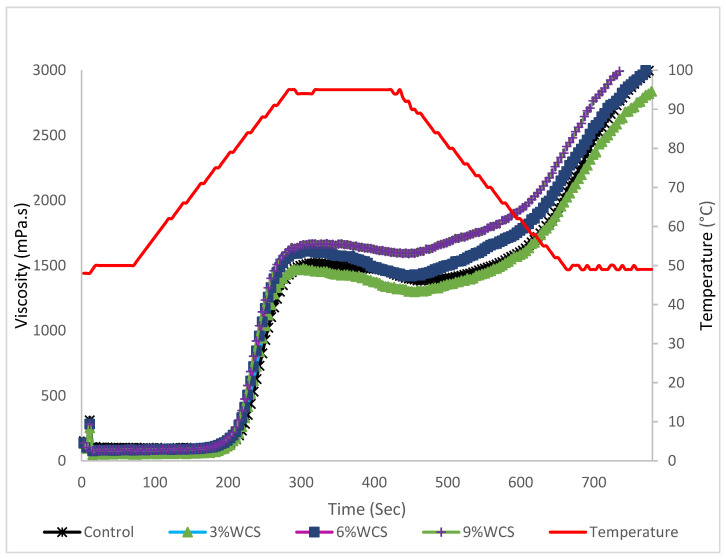
Pasting curves of whole maize meal fortified with WCS. Presented are means with standard deviations.

The pasting properties of maize flours fortified with DCF are presented in Table 3 while pasting curves of maize flours fortified with WCS are presented in Figure 1.

Peak viscosity ranged between 1477–1763 mPa.s. A significant difference was observed at 6% and 9% substitution level of both DCF and WCS. A significant difference was also observed in trough viscosity for samples that had 6% and 9% DCF and 9% WCS, where 9% defatted chia flour formulation had the highest value of 1714 mPa.s. Breakdown viscosity ranged between 49–205 mPa.s, where 9% DCF was the only formulation that had the significantly lower breakdown viscosity as compared to control. However, there was no significant difference observed in final viscosity for all the samples when compared to control (*p* < 0.05). Setback viscosity ranged from 1117–1714 mPa.s. Setback viscosity was generally statistically lower for all the samples with DCF compared to control, whilst samples with whole chia seeds did not have a significant difference. For time to peak, a significant difference was only observed at 9% substitution level of DCF (6.47 s) as compared to control that took 5.35 s. Pasting temperature ranged from 52.33–81.03 °C. A decrease in pasting temperature occurred with an increase in the substitution level of DCF flour and all were significantly different from control when compared at *p* < 0.05. However, whole chia seeds did not influence the pasting temperatures of the flour. The maximum viscosity attained while heating at 95 °C is known as peak viscosity [38]. As explained by [27], peak viscosity is a metric for starch’s ability to hold onto water in terms of inflated granule resistance. Additionally, when the granule structure is no longer able to maintain continuous enlargement, it marks the beginning of granule disruption. Thus the high peak viscosities observed at 6% and 9% substitution level of both DCF and WCS affirms the increasing water-holding capacity of the flour formulation as chia seeds are added. A similar trend was observed in this study on WAC. This could be due to increased bioactive compounds [30] as well as the protein and fiber content contributed by chia seeds [31]. This finding agrees with the observation made in chia–barley composites where chia composites had higher pasting viscosities compared to barley [39]. However, it disagrees with the findings made in a study where partly defatted chia flour (PDCF) was used in wheat muffins and all samples that had PDCF showed significantly lower viscosities when compared with those without PDCF [40].

The lowest viscosity that may be reached during heating at 95 °C is called trough viscosity, and it gauges how the swollen starch granules behave when heated and sheared [41]. Breakdown viscosity can be defined as the difference between the peak and trough viscosities. During breakdown, swollen starch granules rupture and the linear granules leach into the solution, thus, meaning a decreased viscosity [27,42]. The observation made in this study of increasing trough viscosity with decreasing breakdown viscosity with increase in substitution level of defatted chia seeds and whole chia seeds shows that flour formulations with 6% and 9% DCF as well as 9% WCS significantly withstand heating. Leaching of the ruptured starch granules into the solution to lower the viscosity is prevented by the gel formed by chia seeds from the mucilage that forms when they are in contact with water and DCF had a significant impact at 6% level. The observation made in this study agrees with the findings made in a study that involved incorporation of chia seeds in rice flour that led to increased trough viscosity [43].

The viscosity measured at the end of the pasting cycle, known as the final viscosity, indicates how well the starch in flour can produce a viscous paste as cooling occurs [42] due to restructuring and retro gradation of starch granules [38]. In this study, none of the samples significantly differed from control at *p* < 0.05. The ability of the amylose present in the paste to re-associate when the temperature drops is shown by setback viscosity, which is the difference between peak viscosity and final viscosity [44]. The reduction in the setback viscosity with addition of DCF flour could be attributed to the decreasing lipid content, which has been reported to increase the setback viscosity [43]. Low setback viscosity indicates slow retrogradation process, which results in slow staling as well as softer products [27].

Time to peak displays how long it took a sample to reach its maximum viscosity. The lowest time taken by the flour formulation with 9% DCF could be attributed to the higher fiber content with increased surface area to volume ratio, which absorbs water better to form a paste. The low time to peak and pasting temperature is desired since it is related to low energy input [45]. Pasting temperatures refers to the initial temperature when initial rise in viscosity is observed when starch molecules and proteins start to absorb water and swell [38]. It is an indicator of the lowest temperature at which a product can be cooked. Pasting temperature was significantly lower for all the samples with DCF at all levels when compared to control (*p* < 0.05). This trend agrees with the findings made in a study where incorporation of partly defatted chia flour in wheat for muffin preparation lowered the pasting temperatures [46]. The decrease observed in flour formulations with WCS was, however, not significantly different from control. The lower pasting temperatures point to some ‘‘easiness’’ of the paste to increase viscosity when it is heated. However, DCF does not contain any starch, therefore, it is possible that some other components are having this effect instead. This behavior might be brought on by the solubilization of components in fiber [40].

### 3.4. Proximate Composition

The proximate composition of the raw materials that were used in this study are shown in Table 4. Defatted chia four and whole chia seeds were generally richer in crude ash, crude fat, crude protein, crude fiber, and energy value compared to whole maize meal. 

**Figure 2:**
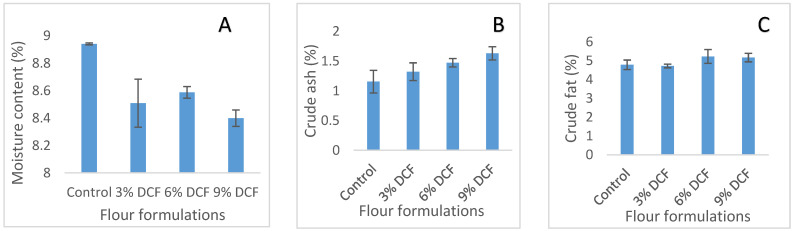

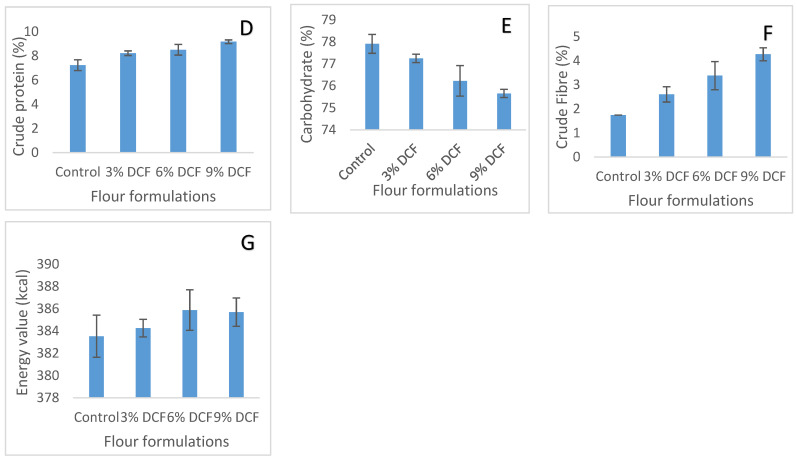
Proximate composition of four formulations fortified with DCF ((**A**), moisture content; (**B**), crude ash; (**C**), crude fat; (**D**), crude protein; (**E**), carbohydrates; (**F**), crude fiber; (**G**), energy value).

The effect of incorporating WCS and DCF on the whole maize meal’s proximate composition is shown in Table 5 and Figure 2, respectively.

Fortification of whole maize meal with both DCF and WCS led to an increase in crude ash, crude protein, crude fiber, and energy values while reducing the carbohydrate level, which was shown to be higher in control sample (77.91%). Moisture content of flour formulations ranged from 8.2–8.94%. Crude ash ranged between 1.15% (control) and 1.63% (9% DCF). Crude fat ranged from 4.78–7.46%, where the 9% WCS formulation had the highest crude fat. Crude protein ranged from 7.22–9.16%, where 9% DCF recorded the highest value. Carbohydrates ranged between 74.69–77.91%, where the control sample recorded the highest level, while crude fiber ranged between 1.74–4.27%, which was highest at 9% level of substitution with DCF. Energy value of the flour formulations used in this study ranged between 383.52 kcal (control) to 398.69 kcal (9% WCS). Fortification with both DCF and WCS generally improved the crude ash, protein, fat, and fiber as well as energy value at all levels of substitution. The findings in this study of the effect that defatted chia flour had on the proximate composition of the flour formulation agrees with the report in [40], who also reported that using by-product of chia oil extraction improved the proximate composition of wheat muffins. While the reported crude fat was higher in whole chia seeds, moisture content, crude ash, and crude protein are comparable with findings in the literature [47,48]. These researchers also reported improved proximate composition in yoghurt and wheat bread with the incorporation of whole chia seeds. The findings in this study also agree with the results from the chia seeds analysis using spectroscopy [49], the authors of which reported that chia seeds are rich in protein and fat.

### 3.5. Consumer Acceptability

**Figure 3:**
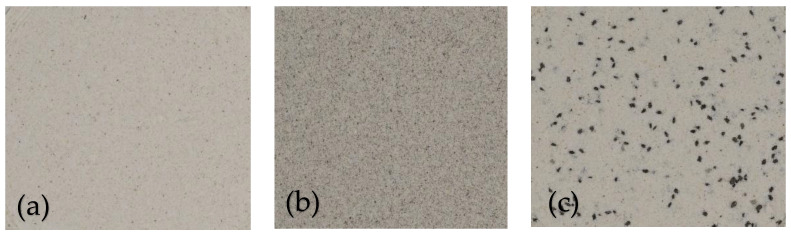
Formulated flour pictures; (**a**)—control; (**b**)—9% DCF; (**c**)—9% WCS.

Results of consumer acceptability test are shown in Table 6. Some images of the formulated flours are presented in Figure 3. Incorporation of both defatted chia flour and whole chia seeds in maize flour did not have significant difference in terms of aroma, taste, mouthfeel, and general acceptability of ugali samples when compared to control at *p* < 0.05.

In terms of color, however, flour fortification with whole chia seeds and defatted chia flour darkened ugali samples but it was not negatively rated by the panelists except at 9% substitution level of defatted chia flour. This could be explained by the total color difference results (∆E*), which show no significant difference for samples with WCS while the ones fortified with DCF show significant difference when compared to control. These results agree with the findings reported by authors that gluten-free breads and wheat breads made with added chia seeds and chia seeds flour are generally acceptable [48,50]. 

## 4. Conclusions

Fortification of whole maize meal with both defatted chia flour and whole chia seeds resulted in improved functional and pasting properties, although it had a negative effect on the color properties. Incorporation of both whole chia seeds and defatted chia seeds flour resulted in nutritionally superior products with high levels of crude ash, crude fat, crude fiber, crude protein, and energy values; the resulting products were generally acceptable to consumers up to 6% substitution level for defatted chia flour while whole chia seeds were generally acceptable to the panelists in all parameters up to 9% level of substitution. Therefore, chia seeds could be exploited as a good fortificant of maize flour for use in making ugali. The substitution level of 9% WCS is recommended for use in ugali preparation since it was nutritionally superior (1.48% crude ash, 7.46% crude fat, 8.20% crude protein, 3.82% crude fiber, and 398 kcal energy value) to control (1.15% crude ash, 4.78% crude fat, 7.22% crude protein, 1.74% crude fiber, and 383 kcal energy value) and still acceptable to the panelists, since the total color difference (∆E*) was not significantly different from control.

## Figures and Tables

**Table 1:** Functional properties of differently formulated flour.

Flours	BD (g/mL)	WAC (mL/g)	OAC (mL/g)	SI (mL/g)	SC (g/g)
Control	0.79 ± 0.0 ^b^	0.78 ± 0.0 ^c^	0.49 ± 0.0 ^bcd^	0.15 ± 0.0 ^f^	2.46 ± 0.1 ^d^
3% DCF	0.81 ± 0.0 ^ab^	0.85 ± 0.1 ^b^	0.55 ± 0.0 ^bc^	0.99 ± 0.2 ^d^	3.05 ± 0.2 ^c^
6% DCF	0.82 ± 0.0 ^a^	0.98 ± 0.0 ^a^	0.58 ± 0.0 ^ab^	2.42 ± 0.2 ^b^	4.85 ± 0.1 ^b^
9% DCF	0.83 ± 0.0 ^a^	0.98 ± 0.0 ^a^	0.67 ± 0.0 ^a^	3.25 ± 0.3 ^a^	5.74 ± 0.5 ^a^
3% WCS	0.79 ± 0.0 ^b^	0.79 ± 0.0 ^c^	0.45 ± 0.1 ^cd^	0.68 ± 0.1 ^e^	2.49 ± 0.3 ^d^
6% WCS	0.75 ± 0.0 ^c^	0.89 ± 0.0 ^b^	0.41 ± 0.0 ^d^	0.91 ± 0.1 ^de^	2.88 ± 0.1 ^cd^
9% WCS	0.72 ± 0.0 ^d^	0.95 ± 0.0 ^a^	0.22 ± 0.0 ^e^	1.31 ± 0.0 ^c^	3.10 ± 0.1 ^c^

Data: Means ± standard deviation (n = 3). There is no significant difference (*p* < 0.05) between the values in a column that has a common superscript letter. Whole chia seeds (WCS), defatted chia seed flour (DCF), BD: bulk density, OAC: oil absorption capacity, SI: swelling index, WAC: water absorption capacity, and SC: Swelling capacity.

**Table 2:** Effect of chia seeds and defatted chia seeds on the color of maize gruel flour.

Sample	L*	a*	b*	Hue Angle	Chroma	∆E*
Control	93.23 ± 0.2 ^a^	−0.45 ± 0.1 ^a^	9.00 ± 0.2 ^a^	92.86 ± 0.8 ^e^	9.01 ± 0.2 ^a^	9.11 ± 0.2 ^d^
3% DCF	89.04 ± 0.5 ^c^	−0.80 ± 0.1 ^bcd^	8.02 ± 0.1 ^c^	95.69 ± 0.7 ^abc^	8.06 ± 0.1 ^c^	11.60 ± 04 ^c^
6% DCF	87.03 ± 1.1 ^d^	−0.83 ± 0.2 ^cd^	7.78 ± 0.3 ^cd^	96.12 ± 1.1 ^ab^	7.83 ± 0.3 ^cd^	13.22 ± 1.1 ^b^
9% DCF	85.34 ± 0.5 ^e^	−0.90 ± 0.2 ^d^	7.56 ± 0.3 ^d^	96.82 ± 1.7 ^a^	7.62 ± 0.3 ^d^	14.66 ± 0.5 ^a^
3% WCS	92.98 ± 0.2 ^a^	−0.63 ± 0.2 ^ab^	8.75 ± 0.2 ^ab^	94.10 ± 1.2 ^de^	8.77 ± 0.2 ^ab^	9.08 ± 0.3 ^d^
6% WCS	92.44 ± 1.0 ^ab^	−0.70 ± 0.1 ^bc^	8.54 ± 0.2 ^b^	94.68 ± 0.8 ^cd^	8.57 ± 0.2 ^b^	9.31 ± 0.7 ^d^
9% WCS	91.54 ± 1.8 ^b^	−0.76 ± 0.1 ^bcd^	8.06 ± 0.2 ^c^	95.38 ± 0.6 ^bcd^	8.10 ± 0.2 ^c^	9.66 ± 1.4 ^d^

Data: Means ± standard deviation (n = 3). Significant differences (*p* < 0.05) exist between values in a column that do not share a superscript letter. DCF: defatted chia seeds flour, WCS: whole chia seeds.

**Table 3:** Pasting properties of different flour formulations.

		Flour Formulations		
Pasting Properties	Control	3% DCF	6% DCF	9% DCF
**PV (mPas)**	1477 ± 54.0 ^c^	1535 ± 53.8 ^bc^	1722 ± 36.8 ^a^	1763 ± 88 ^a^
**TV (mPas)**	1318 ± 75.6 ^c^	1338 ± 37.4 ^c^	1549 ± 63.6 ^ab^	1714 ± 86.0 ^a^
**BV (mPas)**	159 ± 8.2 ^ab^	197 ± 16.5 ^a^	173 ± 10.4 ^ab^	49 ± 4.4 ^c^
**FV (mPas)**	3032 ± 52.7 ^ab^	2877 ± 77.2 ^b^	2830 ± 89.8 ^b^	2832 ± 30.1 ^b^
**SV (mPas)**	1714 ± 96.3 ^a^	1538 ± 42.9 ^b^	1281 ± 26.2 ^c^	1117 ± 98.5 ^c^
**TTPV (min)**	5.35 ± 0.1 ^bc^	4.94 ± 0.3 ^c^	5.72 ± 0.6 ^b^	6.47 ± 0.0 ^a^
**PT (°C)**	81.03 ± 1.1 ^a^	68.93 ± 3.9 ^b^	68.08 ± 0.6 ^b^	52.33 ± 2.2 ^c^

Data: Means ± standard deviation (n = 3). Significant differences (*p* < 0.05) exist between values in a row that do not have the same superscript letter. DCF—defatted chia seeds flour, PV—peak viscosity, TV—trough viscosity, BV—breakdown viscosity, FV—final viscosity, SV—setback viscosity, TTP—time to peak viscosity, and PT—pasting temperature.

**Table 4:** Proximate composition of raw materials.

Raw Material	Moisture Content (%)	Crude Ash (%)	Crude Fat (%)	Crude Protein (%)	CHO (%)	Crude Fiber (%)	Energy Value (kcal)
**DCF**	5.06 ± 0.1 ^b^	6.43 ± 0.4 ^a^	9.19 ± 0.8 ^b^	29.41 ± 0.0 ^a^	49.90 ± 0.9 ^b^	38.26 ± 0.0 ^a^	400.01 ± 5.8 ^b^
**WCS**	5.04 ± 0.0 ^b^	4.78 ± 0.1 ^b^	34.69 ± 1.0 ^a^	21.21 ± 0.0 ^b^	34.286 ± 0.9 ^c^	27.87 ± 0.0 ^b^	534.15 ± 3.9 ^a^
**WMM**	8.94 ± 0.0 ^a^	1.15 ± 0.2 ^c^	4.78 ± 0.2 ^c^	7.22 ± 0.4 ^c^	77.91 ± 0.4 ^a^	1.74 ± 0.0 ^c^	383.52 ± 1.9 ^c^

Data: Means ± standard deviation (n = 3). Values in a column that do not share the same superscript letter are significantly different (*p* < 0.05). DCF—defatted chia seeds flour, WCS—whole chia seeds, WMM—whole maize meal, CHO—carbohydrates.

**Table 5:** Proximate composition of differently formulated flours compared to control.

Sample Name	Moisture Content (%)	Crude Ash (%)	Crude Fat (%)	Crude Protein (%)	CHO (%)	Crude Fiber (%)	Energy Value (kcal)
Control	8.94 ± 0.0 ^a^	1.15 ± 0.2 ^c^	4.78 ± 0.2 ^d^	7.22 ± 0.4 ^d^	77.91 ± 0.4 ^a^	1.74 ± 0.0 ^f^	383.52 ± 1.9 ^d^
3% WCS	8.63 ± 0.4 ^ab^	1.28 ± 0.1 ^bc^	5.58 ± 0.0 ^c^	7.57 ± 1.2 ^cd^	76.94 ± 1.0 ^ab^	2.34 ± 0.4 ^e^	388.25 ± 1.1 ^c^
6% WCS	8.62 ± 0.1 ^b^	1.40 ± 0.1 ^ab^	6.51 ± 0.5 ^b^	8.04 ± 0.0 ^bcd^	75.43 ± 0.5 ^cd^	2.94 ± 0.3 ^cd^	392.48 ± 2.9 ^b^
9% WCS	8.17 ± 0.2 ^c^	1.48 ± 0.1 ^ab^	7.46 ± 0.8 ^a^	8.20 ± 0.0 ^bc^	74.69 ± 0.7 ^d^	3.82 ± 0.1 ^ab^	398.69 ± 4.6 ^a^

Data: Means ± standard deviation (n = 3). Values in a column that do not share the same superscript letter are significantly different (*p* < 0.05). WCS: whole chia seeds, CHO: carbohydrates.

**Table 6:** Consumer acceptability of ugali made from different flour formulations.

Sample	Color	Aroma	Taste	Mouthfeel	General Acceptability
Control	8.16 ± 1.0 ^a^	7.83 ± 1.3 ^a^	7.16 ± 1.5 ^a^	7.50 ± 1.2 ^a^	7.80 ± 0.7 ^a^
3% DCF	7.33 ± 1.2 ^ab^	7.50 ± 1.0 ^a^	7.7 ± 0.8 ^a^	7.50 ± 1.0 ^a^	7.00 ± 1.2 ^a^
6% DCF	6.83 ± 1.2 ^ab^	7.33 ± 0.8 ^a^	6.83 ± 1.3 ^a^	7.00 ± 0.9 ^a^	7.18 ± 0.9 ^a^
9% DCF	6.33 ± 1.5 ^b^	7.00 ± 1.4 ^a^	6.83 ± 1.9 ^a^	7.00 ± 1.2 ^a^	6.67 ± 1.5 ^a^
3% WCS	7.00 ± 1.0 ^ab^	6.68 ± 1.9 ^a^	6.00 ± 1.9 ^a^	6.16 ± 1.8 ^a^	6.83 ± 1.2 ^a^
6% WCS	6.83 ± 1.2 ^ab^	7.00 ± 1.8 ^a^	6.83 ± 1.8 ^a^	6.33 ± 1.6 ^a^	7.00 ± 1.3 ^a^
9% WCS	7.00 ± 1.8 ^ab^	7.18 ± 1.2 ^a^	6.00 ± 1.4 ^a^	6.00 ± 1.0 ^a^	6.67 ± 1.3 ^a^

Data: Means ± standard deviation. Values in a column that do not share the same superscript letter are significantly different (*p* < 0.05). DCF: defatted chia seeds flour, WCS: whole chia seeds.

## Data Availability

The original contributions presented in the study are included in the article, further inquiries can be directed to the corresponding author.
